# Rare clinical presentations of paracoccidioidomycosis: Epidemiological, clinical, and microbiological insights from a Brazilian reference center

**DOI:** 10.1016/j.bjid.2026.105825

**Published:** 2026-06-01

**Authors:** Lucas Castello Agrizzi, Wdson Luis Lima Kruschewsky, Simone Bravim Maifrede, Marco Antônio Urbano Nogarol, Zoilo Pires de Camargo, Anderson Messias Rodrigues, Tânia Regina Grão-Velloso, Paulo Mendes Peçanha, Aloísio Falqueto, Sarah Santos Gonçalves

**Affiliations:** a Universidade Federal do Espírito Santos, Hospital Universitário Cassiano Antônio Moraes, Vitória, ES, Brazil; b Hospital das Clínicas da Faculdade de Medicina da Universidade de São Paulo, Departamento de Doenças Infecciosas e Parasitárias, São Paulo, SP, Brazil; c Universidade Federal do Espírito Santo, Centro de Investigação em Micologia Médica, Departamento de Patologia, Vitória, ES, Brazil; d Universidade Federal do Espírito Santo, Centro de Investigação em Micologia Médica, Programa de Pos-Graduação em Doencas Infecciosas, Vitória, ES, Brazil; e Universidade Federal de São Paulo, Departamento de Microbiologia, Imunologia, e Parasitologia, Laboratório de Emergência de Fungos Patogênicos, São Paulo, SP, Brazil; f Universidade Federal do Espírito Santo, Departamento de Odontologia Clínica, Vitória, ES, Brazil

**Keywords:** *Paracoccidioides*, Paracoccidioidomycosis, Eyelid, Genital, Osteoarticular, Rare presentations

## Abstract

**Background:**

Paracoccidioidomycosis (PCM) is an endemic systemic mycosis in Latin America, caused by species of the Paracoccidioides brasiliensis complex. Involvement of the eyelid, genital, and osteoarticular systems in PCM is rare.

**Objectives:**

This study describes the epidemiological, clinical, and microbiological characteristics of rare presentations of PCM involving the eyelids, genital system, and osteoarticular system in patients treated at a reference center in southeastern Brazil.

**Methods:**

A retrospective cross-sectional analysis was conducted among 33 patients diagnosed with uncommon forms of PCM, proven or probable, between 1973 and 2020. Demographic, clinical, and therapeutic data were retrieved from medical records.

**Results:**

Between 1973 and 2020, 33 patients with rare forms of PCM were identified in Espírito Santo, Brazil, involving the eyelid (*n* = 11), genital (*n* = 10), and osteoarticular (*n* = 12) systems. Most patients were male and had rural exposure. Eyelid and genital involvement occurred in middle-aged men with chronic disease and frequent pulmonary association, with ulcerative lesions predominating. Osteoarticular cases affected younger patients with acute/subacute forms and systemic symptoms. Radiological findings mainly included osteolytic lesions. Histopathology showed high diagnostic yield, while serology was frequently positive. Treatment varied by severity, and relapses were associated with irregular therapy.

**Conclusions:**

These findings expand the clinical spectrum of PCM and highlight the importance of recognizing atypical localizations that may mimic neoplastic or other infectious diseases. Awareness of these rare manifestations, particularly in endemic areas, can improve diagnostic accuracy and patient outcomes.

## Introduction

Paracoccidioidomycosis (PCM) is an endemic systemic mycosis in Latin America, caused by species of the *Paracoccidioides brasiliensis* complex (*P. brasiliensis sensu stricto, P. americana, P. restrepiensis*, and *P. venezuelensis*) and *Paracoccidioides lutzii*.^1^^,^^2^ These are thermodimorphic fungi that grow as filamentous mycelia at 25–28 °C and as yeasts at 35–37 °C or during parasitism in host tissues.^3^ Human infection occurs through the inhalation of fungal propagules. In nature, these microorganisms act as soil saprophytes, typically found in humid environments with dense vegetation cover.^4^

Brazil accounts for approximately 80% of all PCM cases worldwide, with most originating from the South, Southeast, and Midwest regions. The estimated incidence in the country ranges from 1 to 4 cases per 100,000 inhabitants per year.^5^ However, rates are considerably higher in areas of recent colonization, such as the Amazon region, where they range from 9 to 40 cases per 100,000 inhabitants, and have increased more recently in the states of Tocantins, Pará, and Maranhão.^2^^,^^6^, ^7^, ^8^ The actual burden of PCM in Brazil remains uncertain, as the disease is not compulsorily reported to the national notification system. Consequently, most available data derive from epidemiological surveys, case series, and hospital records.^9^, ^10^, ^11^, ^12^, ^13^

The latency period of *Paracoccidioides* spp. in humans is referred to as PCM infection. When the microorganism is reactivated and begins to cause tissue damage, exhibiting characteristic clinical signs and symptoms, the condition is referred to as PCM disease.^4^ Paracoccidioidomycoses can present in two primary clinical forms. The most common form is the chronic form, typically occurring in adults aged 30 to 60. It is characterized by predominant pulmonary involvement, often associated with mucocutaneous or cutaneous lesions, and may also progress to compromise the central nervous system and other organs. The second form is the acute/subacute, or juvenile, type, which usually affects children, adolescents, and young adults under 30. This form follows a rapid, disseminated course, characterized by fever, localized or generalized lymphadenopathy, hepatosplenomegaly, gastrointestinal manifestations, skin and bone lesions, and limited or absent pulmonary involvement.^4^^,^^14^

Involvement of the eyelid, genital, and osteoarticular systems in PCM is rare. Most reports of these atypical presentations are limited to isolated case reports, and such sites are consistently underrepresented in large case series. The present study reports a case series of PCM with rare manifestations ‒ specifically eyelid, genital, and osteoarticular involvement ‒ treated at a PCM referral center in the southeastern region of Brazil.

## Methods

### Study design and participants

This retrospective cross-sectional study included patients with rare forms of PCM (eyelid, genital, and osteoarticular involvement) treated between January 1973 and December 2020 at a referral center for PCM in southeastern Brazil. Cases were identified from the mycological and serological databases of the center, which is responsible for the diagnosis and longitudinal follow-up of PCM patients, who are routinely monitored until completion of antifungal therapy.

Proven cases are characterized by patients with compatible clinical manifestations and by evidence of *Paracoccidioides* spp. in secretions, body fluids, or lesions, as demonstrated by direct mycological examination, culture, or histopathology. Probable cases are defined by suggestive clinical manifestations and the presence of specific antibodies in serum, without evidence of fungal structures or positive cultures in clinical samples.^4^ Serological diagnosis was performed using the double immunodiffusion assay with *P. brasiliensis* antigen (B-339), following standard protocols.^3^

Data were extracted from medical records and included age, sex, ethnicity, occupation, geographic origin (country/state), comorbidities, clinical form, organ involvement, diagnostic methods, and antifungal therapy. The selection of rare sites was based on their low frequency in large case series, their clinical relevance, and their limited representation in the literature, where they are predominantly described in isolated case reports.

### Ethics statement

The authors confirm that this study was conducted in accordance with the ethical standards outlined in the journal’s author guidelines. Ethical approval was obtained from the Ethics Committee (Process number: CAAE: 48798521.3.0000.5060).

## Results

Between 1973 and 2020, 33 patients with rare forms of PCM (eyelid, genital, and osteoarticular) were diagnosed in the state of Espírito Santo, Brazil.

Geographically, most patients originated from rural regions of Espírito Santo, particularly the Southwest Serrana, Central-West, Northwest, and Central Serrana microregions. However, most patients resided in the Metropolitan region at the time of diagnosis, likely reflecting rural-to-urban migration patterns (Table 1).

**Table 1:** Epidemiological characteristics of 33 patients with rare forms.

Variables		Acute Form *n* = 8 (%)	Chronic Form *n* = 25 (%)
Age	Mean = 39.5		
	Median = 45		
Sex	Male	8 (100)	25 (100)
	Female	0	0
Occupation	Rural workers	2 (25)	21 (84)
	Others	6 (75)	4 (16)
Skin colour (self-reported)	White	2 (25)	5 (20)
	Black	2 (25)	3 (12)
	Brown^a^	4 (50)	15 (60)
Residence in the Espírito Santo state region	Southwest mountain^b^	3	1
	West Center^b^	0	0
	Northwest^b^	0	2
	Northeast	0	1
	Central Mountain^b^	0	2
	South Coast	0	2
	Metropolitan	2	9
	Rio Doce^b^	0	3
	Caparaó^b^	1	0

a Brown refers to individuals classified as ‘Pardo’ according to the Brazilian Institute of Geography and Statistics (IBGE).

b Region’s producers of coffee.

None of the patients was immunocompromised. Systemic arterial hypertension was the most frequently reported condition (*n* = 5, 15.2%), followed by schistosomiasis (*n* = 4, 12.0%) and other helminth infections (*n* = 4, 12.0%); tuberculosis (*n* = 2, 6.0%) and diabetes mellitus (*n* = 1, 3.0%) were less common.

### Eyelid

We identified 11 cases of PCM with eyelid involvement over 47-years. All patients were male and presented with the chronic form of the disease, with a mean age of 45.9-years. Most had a history of rural occupational exposure (*n* = 9, 81.8%), and a high proportion were smokers (*n* = 8, 72.7%) and alcohol users (*n* = 8, 72.7%) (Table 1). Regarding self-reported race, 7 patients (63.6%) were brown, 2 (18.2%) were black, 1 (9.1%) was white, and data were unavailable for 1 patient (9.1%).

**Fig. 1:**
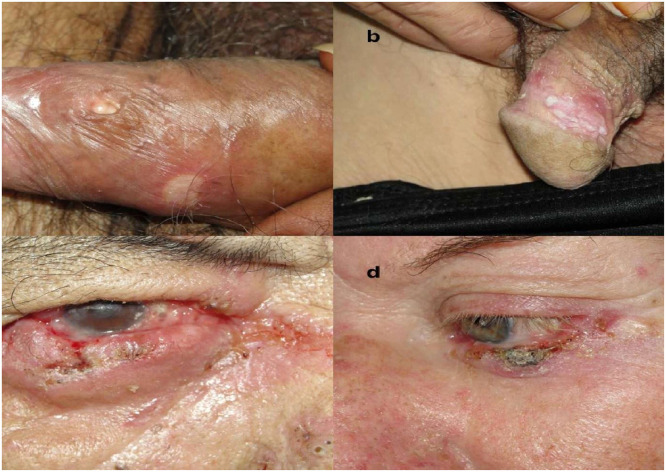
(A) Ulcerated lesions on the prepuce; (B) Vegetative, whitish lesions with an erythematous halo in the balanopreputial sulcus; (C) Eyelid involvement of paracoccidioidomycosis with an extensive lesion on the lower eyelid, presenting a granulomatous, infiltrative, and hemorrhagic aspect, with conjunctival involvement; and (D) Crusted lesion on the lower eyelid. All patients were treated at the Reference Center.

This involvement was frequently associated with additional cutaneous lesions (*n* = 10, 90.9%) and respiratory symptoms (*n* = 8, 72.7%). The description of eyelid lesions was available for 8 patients (72.7%): ulceration was the most frequent presentation (*n* = 5, 62.5%), followed by verrucous lesions (*n* = 2, 25.0%) and papules (*n* = 1, 12.5%). Additionally, 2 patients (18.2%) had conjunctival involvement, described as moriform hyperemia. Some lesions are shown in Fig. 1C and 1D Other reported clinical manifestations included lymphadenomegaly (*n* = 6, 54.5%), oral mucosa lesions (*n* = 6, 54.5%), weight loss (*n* = 5, 45.4%), and fever (*n* = 4, 36.4%).

Histopathological analysis demonstrated a 100% diagnostic yield, while direct microscopy of lesion samples was positive in 80.0% of cases (Table 2). Serology was performed in 8 patients, with a positivity rate of 87.5%.

**Table 2:** Clinical characteristics, diagnosis and treatment of 33 patients.

Variables		Eyelid form, *n* = 11 (%)	Genital form, *n* = 10 (%)	Osteoarticular form, *n* = 12 (%)
**Risk Factors**	Alcoholism	8 (72.7)	8 (80)	4 (33.3)
	Smoking	8 (72.7)	7 (70)	5 (41.7)
**Involved organs**	Lymph nodes	6 (54.5)	5 (50)	8 (66.7)
	Lung	8 (72.7)	10 (100)	5 (41.7)
	CNS	0	1 (10)	0
	Skin lesions	10 (90.9)	6 (60)	8 (66.7)
	Oral mucosa lesions	6 (54.5)	8 (80)	1 (8.3)
	Larynx	3 (27.3)	3 (30)	0
**Diagnostic Methods**				
PCM serology (DID)	Reactive	7 (87.5)	5 (62.5)	8 (88.9)
	Non-reactive	1 (12.5)	3 (37.5)	1 (11)
	NP	3	2	3
Histopathology	Positive	5 (83.3)	10 (100)	10 (83.3)
	NA	1 (16.7)	0	2 (16.7)
	NP	5	0	0
Direct mycological examinations	Positive	4 (80)	3 (75)	3 (60)
	Negative	1 (20)	1 (25)	2 (40)
	NP	6	6	5
PCM treatment	Sulfamethoxazole/ Trimethoprim	9 (81.8)	5 (50)	8 (66.7)
	Amphotericin B	2 (18.2)	5 (50)	10 (83.3)
	Itraconazole	1 (9.1)	1 (10)	4 (33.3)
	Ketoconazole	1 (9.1)	1 (10)	0

NP, No Performed; NA, No Available; CNS, Central Nervous System.

Treatment was also analyzed by affected organ (Table 2). Among patients with eyelid involvement, the most frequently used regimen was trimethoprim–sulfamethoxazole (*n* = 9, 81.8%), followed by amphotericin B (*n* = 2, 18.2%), while itraconazole and ketoconazole were each used in 9.1% of cases (*n* = 1).

### Genital

We identified 10 cases of PCM with genital involvement over 47-years. Similar to eyelid involvement, all patients were male and presented with the chronic form of the disease, with a mean age of 48.2-years. Rural occupational exposure was universal (*n* = 10, 100%), and the majority reported alcohol use (*n* = 8, 80.0%) and smoking (*n* = 7, 70.0%) (Table 1). In this group, 5 patients (50.0%) were brown, 3 (30.0%) were white, 1 (10.0%) was black, and data were unavailable for 1 patient (10.0%).

Lung involvement was observed in all patients (*n* = 10, 100%), while oral mucosa (*n* = 8, 80.0%) and cutaneous involvement (*n* = 6, 60.0%) were present in most cases. Ulceration with erythematous borders and a necrotic center was observed in two cases, while a nodule and a fistula were each reported in one case. Fever was uncommon in cases with genital involvement (*n* = 3, 30%). No single anatomical site predominated; lesions were distributed across the testis, glans, penile base, epididymis, scrotum, anal/perianal region, and balanopreputial sulcus. Representative lesions are shown in Fig. 1A and 1B.

Histopathological examination confirmed the diagnosis in all cases examined, while direct microscopy of lesion samples was positive in 75.0% of cases. Serology was performed in 8 patients, with a positivity rate of 62.5%.

In genital involvement, treatment was more evenly distributed, with trimethoprim-sulfamethoxazole and amphotericin B both used in 50.0% (*n* = 5) of patients, and itraconazole and ketoconazole each administered in 10.0% (*n* = 1) of cases.

### Osteoarticular

We identified 12 cases of PCM with osteoarticular involvement over 47-years. Patients in this group were younger, with a mean age of 29.7-years, and most (*n* = 8, 66.7%) presented with the acute/subacute form. Rural occupational exposure was reported only in 4 cases (33.3%), while the majority (*n* = 8, 66.7%) had other occupations. Alcohol use (*n* = 4, 33.3%) and smoking (*n* = 5, 41.7%) were less frequently reported (Table 1). Regarding race, 7 patients (58.3%) were brown, 3 (25.0%) were white, and 2 (16.7%) were black.

Clinical manifestations of osteoarticular PCM included weight loss (*n* = 9, 75.0%), fever (*n* = 7, 58.3%), and local pain (*n* = 7, 58.3%). Local edema, hyperemia, and fistula formation were not observed. Cutaneous and lymph node involvement were frequent (*n* = 8, 66.7%), whereas lung involvement was less common (*n* = 5, 41.7%). Radiological findings were available for 10 patients (83.3%): osteolytic lesions were observed in 6 cases (60.0%), while expansive lesions (*n* = 2, 20.0%) and synovitis (*n* = 2, 20.0%) were less frequent. The most frequently affected sites were: vertebrae (*n* = 5, 41.7%), knees (*n* = 3, 25.0%), and scapulae (*n* = 3, 25.0%). Other sites included ribs, clavicles, shoulders, fingers, and isolated cases involving long bones and the axial skeleton.

In acute osteoarticular PCM (*n* = 8), serology was performed in all patients, with all tests returning positive results. Histopathology was performed in 6 acute cases, all of which were positive. Direct microscopy of lesion samples was performed in 5 cases, with 3 positive results (60.0%). In chronic osteoarticular cases (*n* = 4), histopathology was the main diagnostic method, performed in all patients with 100% positivity, whereas serology was performed in only one case and was negative (Table 2).

For osteoarticular disease, amphotericin B was the most commonly used agent (*n* = 10, 83.3%), followed by trimethoprim-sulfamethoxazole (*n* = 8, 66.7%) and itraconazole (*n* = 4, 33.3%). Ketoconazole was not used in this group.

Overall, more severe presentations, particularly in osteoarticular cases, were more frequently managed with amphotericin B, whereas trimethoprim-sulfamethoxazole remained the most commonly used therapy across all groups.

### Treatment and outcomes

The study also assessed treatment regularity and disease recurrence. Among the 33 patients, information on treatment adherence was unavailable for 16. Of the remaining 17 patients, 5 received regular treatment, and 12 had irregular therapy. Among those with regular treatment, only 1 relapse was observed (20.0%). In contrast, 6 relapses occurred among patients with irregular treatment, indicating that only half remained disease-free. However, detailed information on dosage and therapy duration was not consistently available in the medical records.

## Discussion

In this retrospective study, we analyzed 33 cases of rare PCM presentations involving the eyelids, genital system, and osteoarticular system. Our findings corroborate previously described epidemiological associations for PCM,^11^^,^^15^ and further delineate patterns specific to these underrecognized clinical manifestations, which are often associated with delayed diagnosis.

The mechanisms underlying the localization of *Paracoccidioides* spp. in unusual sites remain unclear. Hematogenous dissemination from a primary pulmonary focus is the most accepted explanation, supported by the frequent detection of pulmonary lesions in genital and eyelid PCM.^10^^,^^16^ In osteoarticular PCM, both hematogenous spread and direct extension from adjacent foci have been proposed.^17^ The finding that all genital cases had concomitant pulmonary involvement reinforces this hypothesis and suggests that the genital lesions likely represent secondary dissemination rather than primary inoculation.

Most published cases of eyelid involvement occur in middle-aged male smokers with the chronic form of the disease and concomitant lung involvement. Active lesions range from localized erythematous patches with madarosis to ulcerative lesions, without a predilection for any specific site.^15^^,^^18^^,^^19^ In our study, ulceration was the most frequent clinical manifestation. Given its granulomatous and ulcerative presentation, eyelid PCM may mimic neoplastic or other infectious conditions, such as leishmaniasis, sporotrichosis, and tuberculosis, often leading to diagnostic delay or unnecessary surgical procedures.^18^ Diagnosis can be established by culture, direct microscopy, or histopathological analysis, with serological methods demonstrating good accuracy for both diagnostic support and monitoring cure.^20^ With antifungal treatment, lesions typically progress to fibrosis.^15^

Similarly to eyelid involvement, genital PCM is more frequent in middle-aged males with the chronic form of the disease. A review of 52 cases of urogenital PCM showed that over 90% occurred in males with the chronic form; local edema and pain, typically associated with predominantly ulcerative genital lesions, were the most common manifestations, with a median time from symptom onset to diagnosis of 24-weeks.^21^ Our data showed that male sex, chronic form, and lung involvement were consistent findings in all patients with the genital form. Those findings align with Severo et al.,^10^ who reported lung infiltrates on chest radiographs in all 11 cases of genital PCM. In contrast to our findings, in which no single site predominated, the testicle appears to be the most common site in genital PCM, followed by the prostate and penis.^21^

In contrast, osteoarticular involvement is more often reported in a different patient profile: younger individuals with the acute/subacute form of the disease and less frequent lung involvement. Weight loss, fever, and local pain were the most common signs and symptoms, and unifocal osteomyelitis was the predominant clinical manifestation.^21^ Radiological studies, including bone radiography, computed tomography, and magnetic resonance imaging, were performed in cases of suspected osteoarticular PCM. Bone lesions in PCM have been well described in the literature, characterized by sharply defined osteolytic lesions without marginal sclerosis and typically without a periosteal reaction.^8^^,^^17^^,^^22^ These radiological features are essential for distinguishing PCM from neoplastic and pyogenic osteomyelitis processes.

Although histopathology was frequently employed and remains a valuable diagnostic approach in PCM, it should not be regarded as the most reliable method in all clinical settings. Less invasive techniques, such as serology and direct microscopy, also play a crucial role and may provide accurate results when performed by trained professionals.^23^ In many healthcare institutions, however, the limited availability of serological assays or specialized microscopy often leads to the preferential use of biopsy, despite its invasive nature. The turnaround time and cost-effectiveness of each method vary depending on local resources: biopsy may be more accessible or financially viable in specific hospitals, while serological tests can yield faster results when available. Direct microscopy, although inexpensive, requires considerable expertise for precise interpretation and remains highly dependent on operator skill.^23^ In cases with inconclusive histopathological findings, these complementary methods are essential for establishing a diagnosis, particularly for mucocutaneous lesions where biopsy is seldom performed.^23^^,^^24^

Antifungal treatment with amphotericin B, trimethoprim-sulfamethoxazole, or itraconazole should follow the Brazilian guideline recommendations.^4^ In our series, more than half of the patients required hospitalization and intravenous antifungal therapy, reflecting delayed diagnosis and the severity of these rare disease presentations. Paracoccidioidomycoses often require therapy extending beyond 18-months, with periodic serological monitoring to detect early relapse.^4^^,^^5^^,^^14^ The higher relapse rate observed among patients with irregular therapy reinforces the need for long-term follow-up and patient education regarding treatment adherence. Adherence difficulties are common in low-resource settings, where prolonged treatment and drug availability remain significant barriers.^25^^,^^26^

This study has limitations inherent to retrospective medical record analyses, including incomplete or inaccurate data. Nevertheless, it provides epidemiological, clinical, and microbiological insights into rare forms of PCM. Our findings underscore the importance of considering PCM as a differential diagnosis in endemic regions, particularly in the context of diseases such as leishmaniasis, sporotrichosis, tuberculosis, and skin neoplasms. These analyses also highlight trends in the clinical presentation of PCM, which can guide diagnostic reasoning and support a more accurate recognition of conditions that remain underexplored in the current literature.

## Conclusion

In conclusion, our findings provide additional insight into the clinical spectrum of PCM in Brazil, particularly regarding rare presentations involving the eyelids, genital system, and osteoarticular system. These results highlight the importance of considering PCM in the differential diagnosis of atypical manifestations in endemic areas. Increased awareness of these uncommon presentations may contribute to earlier recognition and more accurate diagnosis. Furthermore, this study adds to the current understanding of the epidemiological and clinical characteristics of PCM in southeastern Brazil.

## Conflicts of interest

The authors declare no conflicts of interest.
